# Impacts of COVID-19 on the Life and Work of Healthcare Workers During the Nationwide Partial Lockdown in Vietnam

**DOI:** 10.3389/fpsyg.2021.563193

**Published:** 2021-08-19

**Authors:** Quan Thi Pham, Xuan Thi Thanh Le, Tam Chi Phan, Quang Nhat Nguyen, Nhung Kim Thi Ta, Anh Ngoc Nguyen, Thao Thanh Nguyen, Quynh Thi Nguyen, Huong Thi Le, Anh Mai Luong, David Koh, Men Thi Hoang, Hai Quang Pham, Linh Gia Vu, Trang Ha Nguyen, Bach Xuan Tran, Carl A. Latkin, Cyrus S. H. Ho, Roger C. M. Ho

**Affiliations:** ^1^Institute for Preventive Medicine and Public Health, Hanoi Medical University, Hanoi, Vietnam; ^2^School of Pharmacy, University of Southern California, Los Angeles, CA, United States; ^3^Institute for Global Health Innovations, Duy Tan University, Da Nang, Vietnam; ^4^UFR Biosciences Department, Université Claude Bernard Lyon 1, Villeurbanne, France; ^5^Viet Nam Health Environment Management Agency, Ministry of Health, Hanoi, Vietnam; ^6^Pengiran Anak Puteri Rashidah Sa'adatul Bolkiah (PAPRSB), Institute of Health Science, Universiti Brunei Darussalam, Gadong, Brunei; ^7^Saw Swee Hock School of Public Health, National University of Singapore, Singapore, Singapore; ^8^Faculty of Medicine, Duy Tan University, Da Nang, Vietnam; ^9^Bloomberg School of Public Health, Johns Hopkins University, Baltimore, MD, United States; ^10^Department of Psychological Medicine, National University Hospital, Singapore, Singapore; ^11^Center of Excellence in Evidence-Based Medicine, Nguyen Tat Thanh University, Ho Chi Minh City, Vietnam; ^12^Department of Psychological Medicine, Yong Loo Lin School of Medicine, National University of Singapore, Singapore, Singapore; ^13^Institute for Health Innovation and Technology (iHealthtech), National University of Singapore, Singapore, Singapore

**Keywords:** COVID-19, psychosocial impact, occupational impact, working conditions, healthcare workers, Vietnam

## Abstract

**Background:** Healthcare workers are frontline responders facing a disproportionate increase in occupational responsibilities during the COVID-19 pandemic. Added work-related stress among healthcare personnel may lead to personal and work-related repercussions, such as burnout or decreased quality of care for patients; however, little is known about how the COVID-19 pandemic affects the daily work and life of these workers. This study aimed to evaluate the personal and occupational impacts of the COVID-19 induced partial lockdown in Vietnam among hospital staff.

**Methods:** A cross-sectional web-based study was carried out to collect demographic data and the personal and job impacts of respondents during the second week of national lockdown in April 2020. Snowball sampling technique was applied to recruit 742 hospital staff. The exploratory factor analysis (EFA) was used to examine the validity of the instrument.

**Results:** Of the 742 respondents, 21.2% agreed that “working attitude well-maintained,” followed by 16.1% of respondents who reported that there were “enough employees at work.” Only 3.2% of respondents agreed that “their work was appreciated by society.” Furthermore, healthcare workers in the central region were less likely to have experienced “Avoidance of disclosure and discrimination related to COVID-19” than other areas (Coef. = – 0.25, CI: −0.42 to −0.07). Being women also had a negative association with scores in “Avoidance of disclosure and discrimination related to COVID 19” domain (Coef. = −0.27, CI: −0.43 to −0.12) while having a positive association with “negative attitude towards working conditions” domain (Coef. = 0.19, CI: 0.09 to 0.3). In addition, working in administrative offices (Coef. = 0.20; 95% CI = 0.05 to 0.36) and infectious departments (Coef. = 0.36; 95% CI = 0.09 to 0.63) had a positive association with “Increased work pressure due to COVID 19” domain.

**Conclusion:** These findings revealed marginal impacts of the COVID-19 pandemic on the work and life of hospital staff in Vietnam. Furthermore, this study highlighted the importance of implementing preventive strategies during the nationwide partial lockdown to manage hospital admissions and the burden on healthcare workers. Finally, this study characterizes targeted demographics that may benefit from appreciation by employers and society during a national pandemic.

## Introduction

The WHO has declared the COVID-19 pandemic as a global health emergency (WHO, 2020). As of June 20, 2021, there were 178,965,216 confirmed cases and 3,875,688 deaths across 210 countries, of which the United States (US) had been identified as the hardest hit by this pandemic (Worldometer, 2020). The unprecedented turbulence caused by COVID-19 has crippled health systems worldwide within months and generated tremendous pressure on multiple aspects of the lives of millions of people, particularly healthcare workers (Chew et al., 2020; Tran et al., 2020b). Due to working conditions that require close contact with patients with SARS-CoV-2, the virus that causes COVID-19 and its respiratory transmission mechanism, healthcare workers are more susceptible to SARS-CoV-2 infections. For instance, 20% of medical workers in Italy were infected with the virus, and more than 54 doctors died due to COVID-19 by the end of March. As of April 9, 2020, the Centers for Disease Control and Prevention (CDC) reported approximately 9,282 infections among healthcare workers and 27 deaths in the US (Cdc, 2020).

Understanding the impact of the COVID-19 pandemic among healthcare workers can guide policies and interventions that aim to maintain the attitude and psychological wellbeing of these workers (Konstantinos et al., 2021). Previous studies evaluating health-related effects of the pandemic revealed significantly increased incidence of anxiety and stress within the workforce (Agency, 2020; Huang et al., 2020; Lai et al., 2020; Lima et al., 2020). Regarding working challenges, Schwartz et al. (2020) indicated that, in China, the fear of being infected and work-related pressure were the key motivations for several healthcare workers to find other jobs (Schwartz et al., 2020). In contrast, Chen et al. (2020) showed that SARS-CoV-2 infection was not an immediate concern of healthcare workers, since they had already considered such a scenario in their decision to serve in the hospitals. In addition, the healthcare workers expected that their families would sympathize with their working environment and not be obsessed with the probability of being infected by them; however, healthcare staff admitted that they felt insecure due to the shortage of personal protective equipment (PPE). They also reported feeling helpless when treating severe patients with poor prognoses (Anderson et al., 2020; Chen et al., 2020). Many healthcare staff also expressed their need to have more breaks and better access to PPE. In addition, healthcare workers may require additional training to address situations in which patients refuse to isolate themselves in the hospitals or do not comply with medical protocols because of anxiety or lack of knowledge in patients about COVID-19 (Anderson et al., 2020; Chen et al., 2020). Recently, a systematic review found some psychological impacts on healthcare workers; thus, early psychological intervention is needed for protecting healthcare workers against the COVID-19 pandemic (Hooper et al., 2021).

Within the context of Vietnam, at the time of writing this artcile, five hospitals were epicenters of COVID-19 outbreaks (Ministry of Health of Vietnam, 2020b): Bach Mai Hospital, C Da Nang Hospital, VietNam National Cancer Hospital, National Hospital for Tropical Diseases, and Ho Chi Minh Hospital for Tropical Diseases (Vietnam, 2020). Given limited financial and human resources for healthcare, alongside the underdeveloped health infrastructure in Vietnam, Vietnamese healthcare workers might face adversities, including shortage of PPE, increased workload, and added responsibilities (Dang et al., 2020; Tran et al., 2020a). During the nationwide partial lockdown, healthcare workers spent more time at the hospitals, which might cause a lack of contact with their families, isolation, burnout, frustration, and discrimination (Dang et al., 2020; Kang et al., 2020a; Le et al., 2020).

To our knowledge, prior studies assessed the epidemiological prevalence, clinical characteristics of confirmed COVID-19 cases, and challenges in managing health sequelae; however, limited research has been available on the impacts of COVID-19 on the life and work of healthcare workers in Vietnam. Therefore, this study aims to examine how COVID-19 impacts the work-life quality of hospital staff. These findings may provide useful insights for informing future health policies aiming to tailor support for healthcare workers in the fight against this unpredictable pandemic.

## Methods

### Study Design

A cross-sectional, hospital-based survey was carried out during 1 week of nationwide partial lockdown, particularly, from April 7 to 14, 2020, in Vietnam. The rationale for conducting research within this duration was that it overlapped with a full lockdown at Bach Mai Hospital that was implemented to mitigate the transmission of COVID-19. This period was considered a challenging time for hospital staff nationwide, as a leading hospital, equipped with modern and adequate equipment, becoming the largest pandemic cluster in the country. Thus, it was necessary to perform a rapid assessment to capture the impacts of the pandemic on the life and work of this forefront workforce.

### Sample Size and Sampling Method

The snowball sampling technique was applied to recruit respondents. At the beginning of the recruitment process, a core staff group at the Institute of Preventive Medicine and Public Health, Hanoi Medical University was established. By providing the link to the survey through the computers or smartphones of the respondents, the core group was able to access their close contacts or other groups on social media networks, such as Facebook, Zalo applications. The key persons who had been involved in the study were instructed to invite other Vietnamese medical staff to join in the survey. Respondents were recruited according to the following inclusion criteria: (1) agreeing to engage in the research by approving the online informed consent forms, (2) being able to access the questionnaire on an online platform, namely, Surveymonkey, and (3) being able to read and answer the questionnaire. In this study, hospital staff were defined as healthcare workers serving in the hospitals, including doctors, nurses, and administrative staff. A total of 742 hospital staff working in 63 provinces of Vietnam were selected for this study during 1 week of data collection.

### Measure and Instruments

A self-reported questionnaire consisting of 24 questions in the form of single-choice, multiple-choice, and open-ended questions on the effects of the COVID-19 pandemic on the personal lives and works of healthcare staff were sent to the respondents. The questionnaire was developed according to the one used for assessing life and job impacts due to the SARS pandemic (Koh et al., 2005).

#### Demographic Characteristics

We included questions to measure sociodemographic characteristics, such as age, gender (men/women), marital status (single/separated/windowed/married), educational level, and living areas (north/central/south).

#### Occupational Characteristics

Participants were asked about their current title (doctors/ nurses/medical technicians/pharmacists/drivers/receptionists/administrative staff/others) and current work status, including years of experience, working places, and level of the hospitals they served.

#### Information Regarding the Impact of COVID-19 on the Personal Life and Work of Healthcare Workers

To identify the impacts of COVID-19 on the life and work of hospital staff, we asked the respondents to report their experiences related to COVID-19 using 14 multiple-choices questions, namely the following: (1) “I have to do work that I normally do not do”; (2) “I have additional workload”; (3) “I have to work overtime”; (4) “I feel more stressful at work”; (5) “there is conflict among colleagues”; (6) “I have been afraid of telling my family about the risk of exposure to SARS-CoV-2”; (7) “People avoid me because of my job”; (8) “I avoid telling other people about the nature of my job”; (9) “People avoid my family members because of my job”; (10) “my working attitude is not well maintained”; (11) “there are insufficient employees at my workplace to handle the different demands”; (12) “I do not feel appreciated by the hospital/clinic/my employer”; and (13) “I do not feel appreciated by the society.” Each question had five options to respond (1 indicates “strongly disagree,” 2 indicates “slightly agree,” 3 indicates “somewhat agree,” 4 indicates “mostly agree,” and 5 indicates “totally agree”).

Finally, the participants were asked to report their perceptions on the necessity of means of support (food and other necessities, PPE) and the sources of support (family/friends and relatives/colleagues/workplace/government/organizations, and other philanthropists) that they would like to receive.

### Data Analysis

The data were analyzed using STATA 15.0 software (StataCorp LP, College Station, TX, USA). Descriptive statistics were used to report characteristic data covering mean, SD, percentage, and frequency. The exploratory factor analysis (EFA) was applied to assess the construct validity and define interpretable underlying sub-domains of measurement regarding perceived impacts of COVID-19 on the life and work of health workers. We also employed principal component analysis to extract said domains. A threshold defined by the screen test was set at an eigenvalue of 1.5. tTo increase the interpretability of sub-domains of the measurement, we used Orthogonal Varimax rotation with Kaisers' normalization to reorganize items in scales. The minimum factor loading cut-off point of this study was set at 0.43. A cross-loading in one item was performed and then assigned to the appropriate domain according to the overarching dimension and nature of the question. There were three sub-domains identified by EFA, namely the following: (1) avoidance of disclosure and discrimination related to COVID-19 (4 questions), (2) negative attitude towards working conditions (4 questions), and (3) increased work pressure due to COVID-19 (5 questions). Cronbach's alpha described the internal consistency reliability of each domain. Subsequently, we applied a multivariable regression model to identify associated factors within each domain of the EFA. To obtain reduced models, stepwise forward selection strategies were performed with a threshold of log-likelihood ratio test was equal to a *p*-value of 0.2. A *p*-value of < 0.05 was considered statistically significant.

### Ethical Consideration

The research was ethically approved by the Review Committee at the Institute for Preventive Medicine and Public Health, Hanoi Medical University, dated March 28, 2020. The purpose of the research and informed consent was written and obtained online from respondents, who decided to participate. Participation was voluntary, and anonymity was assured. Respondents could decline to participate or withdraw from the online survey at any time.

## Results

The sociodemographic characteristics of the respondents are presented in Table 1. Among 742 respondents who completed the survey, the majority were married (78.7%), living with family or friends (91.9%), and working in the North (71.6%). Approximately two-thirds (65.8%) of the respondents were women; the mean age was 36.3. Regarding occupational characteristics, approximately half of the respondents (51.1%) were doctors, followed by nurses (28.0%), and other titles, including technicians, pharmacists, and receptionists (20.9%); their accumulated working years were 11.4 (SD = 8.8 years) on average. Health staff serving at provincial hospitals and central hospitals accounted for 31.3 and 29.1% of the respondents, respectively.

**Table 1:** Sociodemographic characteristics of respondents.

	***N***	**%**
**Region**
Northern	531	71.6
Central	149	20.1
South	62	8.4
**Gender**
Men	254	34.2
Women	488	65.8
**Marital status**
Single / Separated / Widowed	158	21.3
Married	584	78.7
**Living with**
Family/friends	682	91.9
Alone	60	8.1
**Education**
University and lower	453	61.1
Master/PhD	289	39.0
**Job title**
Doctor	379	51.1
Nurse	208	28.0
Others	155	20.9
**Department**
Emergency-Intensive care	63	8.5
Internal medicine	95	12.8
Surgery-Obstetrics-Pediatrics	91	12.3
Imaging Diagnosis-Scientific laboratory—Clinic	119	16.0
Administrative offices	110	14.8
Infectious disease-Infection control	42	5.7
Preventive medicine-Public Health-Nutrition	86	11.6
Others	136	18.3
**Level of hospital**
Central level	216	29.1
Provincial level	232	31.3
District health center	101	13.6
Others	193	26.0
	**Mean**	**SD**
**Age (years)**	36.3	9.1
**Years of career (years)**	11.4	8.8

Table 2 depicts the construct validity of the questionnaire with respect to the impacts of COVID 19 on the life and work of hospital staff. Three domains, namely “Avoidance of disclosure and discrimination related to COVID 19,” “Negative attitude towards working conditions,” and “Increased work pressure due to COVID 19” were determined from the EFA. The reliability of the three mentioned domains was good, with Cronbach's alpha values being 0.78, 0.80, and 0.81, respectively. Table 2 also presents the proportion of participants who responded “Totally agree” with each item. The highest percentage was for item “Have to do work which never been done” (4.6%), while the item “Working attitude not maintained well” (0%) had the lowest percentage.

**Table 2:** Impact on life and work of respondents due to COVID-19.

**Item**	**Totally agree**		**Avoidance of disclosure and discrimination related to COVID-19**	**Negative attitude towards working conditions**	**Increased work pressure due to COVID- 19**
	***n***	**%**			
Have to do work which never been done	16	4.6			0.83
Have to work overtime	14	4.0			0.87
Increased workload	14	4.0			0.84
Do not dare to tell your family about your risk	12	3.4	0.64		
Being avoided because of work	6	1.7	0.82		
Avoid sharing information about own job	6	1.7	0.74		
Relatives being avoided because of work	5	1.4	0.83		
More stressful at work	4	1.2			0.55
There is conflict between colleagues	3	0.9			0.48
There are not enough employees at work	1	0.3		0.78	
Not be appreciated by the unit leader	1	0.3		0.80	
Not be appreciated by society	1	0.3		0.74	
Working attitude is not maintain well	0	0.0		0.79	
**Cronbach's alpha**			0.78	0.80	0.81
**Mean**			2.7	2.3	2.3
**SD**			0.7	0.5	0.6

Table 3 displays the perception of support provided during the pandemic. The majority of respondents reported that the primary sources of providing them with necessary goods were their family, and friends and relatives (91.7 and 81.7%, respectively). Regarding PPE support, 95.1, 86.3, and 85.1% of respondents agreed that it should be provided by the workplace, the government, and other organizations, respectively. Most of the respondents said that it was necessary to organize morale-building activities to support them in the battle against COVID-19.

**Table 3:** Perception of the support provided during the COVID-19 pandemic.

	***n***	**%**
**Provided with necessary goods**
From family	320	91.7
From friends and relatives	285	81.7
From colleagues	269	77.1
From workplace	289	82.8
From the Government	291	83.4
From organizations and other philanthropists	277	79.4
**Provided with adequate personal protective equipment**
From family	191	54.7
From friends and relatives	183	52.4
From colleagues	221	63.3
From workplace	332	95.1
From the Government	301	86.3
From organizations and other philanthropists	297	85.1
**Organize advocacy activities**	319	91.4

Factors associated with the perception of COVID-19 impact on life and work are presented in Table 4. Being women (Coef. = – 0.27; 95% CI = −0.43 to −0.12), working in the administrative office (Coef. = – 0.29; 95% CI = −0.5 to −0.07) and preventive medicine-public health-nutrition departments (Coef. = −0.32; 95% CI = −0.54 to −0.09) and working in the central region (Coef. = −0.25; 95% CI = −0.43 to −0.07) had a negative correlation with “avoidance of disclosure and discrimination related to COVID-19.” Those who agreed that their friends and relatives were the sources of providing PPE (Coef. = 0.22; 95% CI = 0.07 to 0.38) had a negative association with “avoidance of disclosure and discrimination related to COVID-19,” while receiving PPE from the government had a positive association with this domain (Coef. = −0.29; 95% CI = −0.51 to −0.06).

**Table 4:** Multivariate regression for identifying factors associated with perception on life and job impacts due to COVID-19.

	**Avoidance of disclosure and discrimination related to COVID-19**		**Negative attitude towards working conditions**		**Increased work pressure due to COVID-19**	
	**Coef**.	**95% CI**	**Coef**.	**95% CI**	**Coef**.	**95% CI**
**Region (vs. North)**
Central	−0.25^***^	−0.43; −0.07				
**Gender (vs. men)**
Women	−0.27^***^	−0.43; −0.12	0.19^***^	0.09; 0.30		
**Years of career (years)**	−0.01	−0.01; 0.00				
**Job title (vs. doctor)**
Nurse	0.14	-0.03; 0.32	−0.10	−0.22; 0.02	−0.14^*^	−0.28; 0.01
Others	0.19^*^	−0.01; 0.38				
**Level of hospitals (vs central level)**
District health center	−0.15	−0.37; 0.07			0.12	−0.05; 0.29
Others	−0.14	−0.31; 0.03				
**Marital status (vs. single/separated/widowed)**
Marriage			−0.18^***^	−0.29; −0.06		
**Age**					0.01^***^	0.00; 0.02
**Education (vs. university and lower)**
Master/PhD					−0.17^**^	−0.31; −0.03
**Living with (vs. family/friends)**
Alone					−0.13	−0.33; 0.07
**Department (vs. emergency-intensive care)**
Internal medicine					0.14^*^	−0.03; 0.32
Administrative offices	−0.29^***^	−0.50; −0.07			0.20^**^	0.05; 0.36
Infectious disease-Infection control			0.18	−0.06; 0.42	0.36^**^	0.09; 0.63
Preventive medicine-Public health-Nutrition	−0.32^***^	−0.54; −0.09	0.11	−0.04; 0.27		
Others	−0.19^*^	−0.38; 0.00				
**Provided with necessary goods (agree vs not agree)**
From family	0.23	−0.09; 0.54				
From friends and relatives	−0.24^*^	−0.50; 0.01			−0.29^***^	−0.50; −0.07
From colleagues					0.24^**^	0.03; 0.46
From workplace	0.23^*^	−0.01; 0.47			0.28^***^	0.07; 0.49
**Provided with adequate personal protective equipment**
**(agree vs. not agree)**
From friends and relatives	0.22^***^	0.07; 0.38				
From the Government	−0.29^**^	−0.51; −0.06				
From organizations and other philanthropists					0.15^*^	−0.01; 0.32
**Organized advocacy activities (agree vs. not agree)**			−0.18^***^	−0.24; −0.12		

*** *p < 0.01*,

** *p < 0.05*,

* *p < 0.1*.

Female hospital staff (Coef. = 0.19, 95% CI = −0.09 to 0.3) were associated with increased scores in the “Negative attitude towards working conditions” domain. In contrast, being married (Coef. = −0.18, 95% CI = −0.29 to −0.06) and organizing advocacy activities (Coef. = −0.18, 95% CI = −0.24 to −0.12) were negatively associated with scores in this domain.

Age (Coef. = 0.01, 95% CI = 0.00 to 0.02), working in the administrative office (Coef. = 0.2, 95% CI = 0.05 to 0.36), and the infectious diseases-infection control department (Coef. = 0.36, 95% CI = 0.09 to 0.63), and being provided with necessity goods from the workplace (Coef. = 0.28, 95% CI = 0.07 to 0.49) were factors positively associated with “Increased work pressure due to COVID-19.” Meanwhile, educational achievement being Masters or PhD (Coef. = −0.17, 95% CI = −0.31 to −0.03) and being provided with necessity goods from friends and relatives (Coef. = −0.29, 95% CI = −0.50 to −0.07) were negatively associated with “Increased work pressure due to COVID 19.”

## Discussion

The virus SAR-CoV-2 can be transmitted in different ways, and all populations are susceptible to the virus (Xue-Yan Zhang et al., 2020). Patients suffering from COVID-19 diseases can have mild to life-threatening symptoms, such as acute respiratory symptoms (Aristides Tsatsakis et al., 2020). Neurological complications were also reported among COVID-19 patients (Pennisi et al., 2020).

To our knowledge, this study is among the first to assess the impact of the COVID-19 pandemic on the personal life and work of healthcare staff in Vietnam. Contrary to this hypothesis, however, the results showed that the life and work of healthcare staff were marginally affected by the pandemic. This result might be attributed to vigorous policy and actions of the Vietnamese government to control the pandemic. From these results, we have identified baseline and occupational demographics that need additional morale and employer support during the pandemic.

This study indicated that only 3.4% of respondents did not dare to share the risk of COVID-19 infection with their families, and 1.2% of them suffered from more work-related stress than before. The results contrasted with research in Wuhan, China. In Wuhan, when the COVID-19 epidemic spread, healthcare workers felt stressed and experienced serious mental problems; however, they were less likely to share their problems with their families (Kang et al., 2020b). A possible explanation for this difference is that the Vietnamese government responded rapidly, quarantined infected people, kept their indirect connections under surveillance, and mobilized existing resources at the early stages of the outbreak in January (Tran et al., 2020a,c). These necessary actions by the government minimized the burden on the health system, kept COVID-19 under control, and ultimately reduced the pressure on medical staff at the later stages of the outbreak (Black, 2020).

Noticeably, only 3.2% of respondents agreed that their work was appreciated by society. In Vietnam, healthcare workers often function in high-pressure environments but receive lower income compared with their counterparts in developed countries. The lack of financial incentives might lead healthcare workers to feel that their work is unappreciated by society. This result contrasted with the research of Koh in Singapore during the SARS epidemic (Koh et al., 2005). In Koh's study, 77% of health workers responded that they felt society highly appreciated their works. This finding implied the need for social encouragement towards Vietnamese healthcare workers, especially during the partial lockdown period.

The majority of respondents agreed that being provided necessary goods (by their family) and PPE (by their workplace) would help them overcome additional occupational burden of the pandemic. This positive attitude about COVID-19 was in line with the findings of Huynh Giao et al. (2020). A plausible reason for these results is that Vietnam had recorded more than 200 cases without mortality, and most of the confirmed cases were imported during this survey period (Ministry of Health of Vietnam, 2020a). Providing adequate, necessary goods and support for PPE to health workers were considered as important factors in addressing their concerns about the risk of COVID-19 infection to themselves and their families (Dewey et al., 2020).

Our study indicated that healthcare workers in the central region were less likely to experience “avoidance of disclosure and discrimination related to COVID-19” than those working in other areas. An explanation for this result could be that community spread and confirmed cases of COVID-19 were concentrated in Hanoi and Ho Chi Minh City, two metropolitan cities in the north and the south of Vietnam, respectively. As a result, the risk of infection for health workers in the central region was lower than in other regions.

Female hospital staff had a negative association with scores of “avoidance of disclosure and discrimination related to COVID-19” domain and a positive association with “negative attitude towards working conditions” domain. An explanation for this result could be that women were willing to share their difficulties with others, and therefore, regarded as better adapted to the situation once they disclosed their problems (Derlega and Chaikin, 1976); however, these workers tended to have more negative score associations compared to male men participants. This was similar to the result of Wenham et al. (2020), which showed that women suffered more serious mental challenges than men during the COVID-19 pandemic. A possible reason was that the closure of schools to curb the spread of the virus increased domestic chores and responsibilities for women. Therefore, women healthcare workers had to strive to maintain work–family balance, which might lead to burnout and negative attitudes towards working conditions among women healthcare workers (Alon et al., 2020; Wenham et al., 2020).

Hospital staff face infection risks from both positive and asymptomatic patients because of their close, frequent contacts and longer-than-usual working hours in this pandemic (Li et al., 2003; Shih et al., 2007). Findings in this study showed that healthcare workers working in administrative offices and infectious departments had “Increased work pressure due to COVID-19” compared to those working in the emergency-intensive care department. This finding was understandable in the context of Vietnam, given that COVID-19 patients had mainly mild symptoms, with few severe cases requiring intensive care. As a result, hospital staff working in the emergency-intensive care unit would not be as overloaded as those working at the two mentioned departments. Married healthcare workers and those who agreed to organize advocacy activities were also less likely to have “negative attitude towards working conditions” than other groups. This finding was similar to a study in China, showing that family activities and entertainment increased morale and the quality of life in people struggling with the COVID-19 epidemic (Zhang and Ma, 2020). Our findings implied the need for increased family and social support for healthcare workers during pandemics like COVID-19.

This research has several strengths. One of these strengths is that this study is among the first to evaluate the impact of the COVID-19 pandemic on the personal life and work of health care workers in Vietnam during its first nationwide partial lockdown. Another strength is that this study elucidated factors associated with the personal and occupational impacts of the COVID-19 pandemic. Apart from the strengths mentioned above, this study contained several limitations. First, more than half of the study respondents were doctors and hospital staff that worked in northern Vietnam, suggesting sampling bias since this could not fairly represent the distribution of Vietnamese healthcare workers. Second, participants were recruited *via* a snowball sampling method and the survey was administered as a web-based survey, rather than random selection from a nationally represented sample frame. Third, the survey lasted for only 1 week, and might not fully capture the significant impact of the pandemic on the lives and work of respondents. Fourth, online self-reporting might cause recall bias and social desirability response biases. Overall, a cross-sectional design was unable to identify the longitudinal relationships between associated factors and their outcomes.

## Conclusion

Contrary to previous literature and media anecdotes, this study indicated marginal impacts of the COVID-19 pandemic on the work and life of hospital staff during an unprecedented lockdown in Vietnam. This study also supported the intensive preventive and control measures at the early stages of the pandemic from the Vietnamese government that mitigated transmission of COVID-19 while decreasing the probability of drastic hospital admissions and severe diseases. From these results, we have identified baseline and occupational demographics that need additional morale and employer support during the pandemic. Healthcare workers those who are women, have single marital status, working in a non-central area of Vietnam, and do not work in the emergency-intensive care department should have more support from their employers and community.

## Data Availability Statement

The raw data supporting the conclusions of this article will be made available by the authors, without undue reservation.

## Ethics Statement

The research was ethically approved by the Review Committee at Institute for Preventive Medicine and Public Health, Hanoi Medical University dated 28 March 2020. The patients/participants provided their written informed consent to participate in this study.

## Author Contributions

Conceptualization and writing—review and editing: QP, XL, TP, QN, NT, AN, TN, QN, HL, AL, DK, MH, HP, LV, TN, BT, CL, CH, and RH. Data curation: NT, QN, and TN. Data analysis: QP, XL, LV, and HP. Methodology: QP, XL, HL, AN, DK, and BT. Supervision: XL, HL, BT, AL, and RH. Writing, original draft: QP, XL, TP, and QN. Project administration: QN, MH, and TN. All authors contributed to the article and approved the submitted version.

## Conflict of Interest

The authors declare that the research was conducted in the absence of any commercial or financial relationships that could be construed as a potential conflict of interest.

## Publisher's Note

All claims expressed in this article are solely those of the authors and do not necessarily represent those of their affiliated organizations, or those of the publisher, the editors and the reviewers. Any product that may be evaluated in this article, or claim that may be made by its manufacturer, is not guaranteed or endorsed by the publisher.

## References

[B1] Agency C. I. (2020). The World Factjournal—Vietnam. Central Intelligence Agency. Available online at: https://www.cia.gov/library/publications/the-world-factjournal/geos/vm.html (accessed May 6, 2020).

[B2] Alon T. M. Doepke M. Olmstead-Rumsey J. Tertilt M. (2020). The Impact of COVID-19 on Gender Equality. Cambridge, MA: National Bureau of Economic Research.

[B3] Anderson M. Mckee M. Mossialos E. (2020). Covid-19 exposes weaknesses in European response to outbreaks. BMJ 368, m1075. 10.1136/bmj.m1075 32188590

[B4] Aristides Tsatsakis D. C. Luca F. Et A. (2020). SARS-CoV-2 pathophysiology and its clinical implications: an integrative overview of the pharmacotherapeutic management of COVID-19. Food Chem. Toxicol. 146, 111769. 10.1016/j.fct.2020.111769 32979398 PMC7833750

[B5] Black G. (2020). Vietnam May Have the Most Effective Response to Covid-19. Available online at: https://www.thenation.com/article/world/coronavirus-vietnam-quarantine-mobilization/ (accessed May 2, 2020).

[B6] Cdc (2020). Characteristics of health care personnel with COVID-19—United States, February 12-April 9, 2020. MMWR. Morb. Mortal Wkly. Rep. 69, 477–481. 10.15585/mmwr.mm6915e6 32298247 PMC7755055

[B7] Chen Q. Liang M. Li Y. Guo J. Fei D. Wang L.. (2020). Mental health care for medical staff in China during the COVID-19 outbreak. Lancet Psychiatry 7, e15–e16. 10.1016/S2215-0366(20)30078-X 32085839 PMC7129426

[B8] Chew N. W. S. Lee G. K. H. Tan B. Y. Q. Jing M. Goh Y. Ngiam N. J. H.. (2020). A multinational, multicentre study on the psychological outcomes and associated physical symptoms amongst healthcare workers during COVID-19 outbreak. Brain Behav. Immun. 88, 559–565. 10.1016/j.bbi.2020.04.049 32330593 PMC7172854

[B9] Dang A. K. Le X. T. T. Le H. T. Tran B. X. Do T. T. T. Phan H. T. B.. (2020). Evidence of COVID-19 Impacts on Occupations During the First Vietnamese National Lockdown. Ann. Glob. Health. 86, 112. 10.5334/aogh.2976 32944509 PMC7473180

[B10] Derlega V. J. Chaikin A. L. (1976). Norms affecting self-disclosure in men and women. J. Consult. Clin. Psychol. 44, 376–380. 10.1037/0022-006X.44.3.376 932266

[B11] Dewey C. Hingle S. Goelz E. Linzer M. (2020). Supporting clinicians during the COVID-19 pandemic. Ann. Intern. Med. 172, 752–753. 10.7326/M20-1033 32196544 PMC7106065

[B12] Giao H. Nguyen Thi N. Thi Ngoc Han N. Khanh T. Ngan V. Tam V.. (2020). Knowledge and attitude toward COVID-19 among healthcare workers at District 2 Hospital, Ho Chi Minh City. Asian Pac. J. Trop. Med. 13, 260–265. 10.4103/1995-7645.280396

[B13] Hooper J. J. Saulsman L. Tammy H. Waters F. (2021). Addressing the psychological impact of COVID-19 on healthcare workers: learning from a systematic review of early interventions for frontline responders. BMJ Open 11, e044134. 10.1136/bmjopen-2020-044134 34020974 PMC8142676

[B14] Huang J. Z. Han M. F. Luo T. D. Ren A. K. Zhou X. P. (2020). [Mental health survey of 230 medical staff in a tertiary infectious disease hospital for COVID-19]. Zhonghua Lao Dong Wei Sheng Zhi Ye Bing Za Zhi. 38, E001. 10.3760/cma.j.cn121094-20200219-00063 32131151

[B15] Kang L. Li Y. Hu S. Chen M. Yang C. Yang B. X.. (2020a). The mental health of medical workers in Wuhan, China dealing with the 2019 novel coronavirus. Lancet Psychiatry 7, e14. 10.1016/S2215-0366(20)30047-X 32035030 PMC7129673

[B16] Kang L. Ma S. Chen M. Yang J. Wang Y. Li R.. (2020b). Impact on mental health and perceptions of psychological care among medical and nursing staff in Wuhan during the 2019 novel coronavirus disease outbreak: a cross-sectional study. Brain Behav. Immun. 87, 11–17. 10.1016/j.bbi.2020.03.028 32240764 PMC7118532

[B17] Koh D. Lim M. K. Chia S. E. Ko S. M. Qian F. Ng V.. (2005). Risk perception and impact of Severe Acute Respiratory Syndrome (SARS) on work and personal lives of healthcare workers in Singapore: what can we learn? Med. Care 43, 676–682. 10.1097/01.mlr.0000167181.36730.cc 15970782

[B18] Konstantinos F. Konstantinos Poulasa D. K. Apostolos V. Michalis L. Dimitrios K. Anca Oana D.. (2021). Improved strategies to counter the COVID-19 pandemic: lockdowns vs. primary and community healthcare. Toxicol. Rep. 8, 1–9. 10.1016/j.toxrep.2020.12.001 33294384 PMC7713637

[B19] Lai J. Ma S. Wang Y. Cai Z. Hu J. Wei N.. (2020). Factors associated with mental health outcomes among health care workers exposed to coronavirus disease 2019. JAMA Netw. Open 3, e203976. 10.1001/jamanetworkopen.2020.3976 32202646 PMC7090843

[B20] Le X. T. T. Dang A. K. Toweh J. Nguyen Q. N. Le H. T. Do T. T. T.. (2020). Evaluating the psychological impacts related to COVID-19 of vietnamese people under the first nationwide partial lockdown in Vietnam. Front. Psychiatry 11, 824. 10.3389/fpsyt.2020.00824 32982807 PMC7492529

[B21] Li L. Cheng S. Gu J. (2003). SARS infection among health care workers in Beijing, China. JAMA. 290, 2662–2663. 10.1001/jama.290.20.2662 14645305

[B22] Lima C. K. T. Carvalho P. M. M. Lima I. Nunes J. Saraiva J. S. De Souza R. I.. (2020). The emotional impact of Coronavirus 2019-nCoV (new Coronavirus disease). Psychiatry Res. 287, 112915. 10.1016/j.psychres.2020.112915 32199182 PMC7195292

[B23] Ministry of Health of Vietnam (2020a). Statistics on COVID-19 disease situation Vietnam. Available online at: https://ncov.moh.gov.vn/ (accessed May 5, 2020).

[B24] Ministry of Health of Vietnam (2020b). Updated Information Web Page on Disease COVID−19: Ministry of Health of Vietnam. Vietnam: Ministry of Health of Vietnam. Available online at: https://ncov.moh.gov.vn/ (accessed May 5, 2020).

[B25] Pennisi M. Falzone L. G. Fisicaro F. Ferri R. Bella R. (2020). SARS-CoV-2 and the nervous system: from clinical features to molecular mechanisms. Int. J. Mol. Sci. 21:5745. 10.3390/ijms21155475 32751841 PMC7432482

[B26] Schwartz J. King C. C. Yen M. Y. (2020). Protecting healthcare workers during the coronavirus disease 2019 (COVID-19) outbreak: lessons from Taiwan's severe acute respiratory syndrome response. Clin. Infect. Dis. 71, 858–860. 10.1093/cid/ciaa255 32166318 PMC7108122

[B27] Shih F. J. Gau M. L. Kao C. C. Yang C. Y. Lin Y. S. Liao Y. C.. (2007). Dying and caring on the edge: Taiwan's surviving nurses' reflections on taking care of patients with severe acute respiratory syndrome. Appl. Nurs. Res. 20, 171–180. 10.1016/j.apnr.2006.08.007 17996803 PMC7127079

[B28] Tran B. X. Hoang M. T. Pham H. Q. Hoang C. L. Le H. T. Latkin C. A.. (2020a). The operational readiness capacities of the grassroots health system in responses to epidemics: implications for COVID-19 control in Vietnam. J. Glob. Health 10, 011006. 10.7189/jogh.10.011006 32566168 PMC7294390

[B29] Tran B. X. Hoang M. T. Vo L. H. Le H. T. Nguyen T. H. Vu G. T.. (2020b). Telemedicine in the COVID-19 pandemic: motivations for integrated, interconnected, and community-based health delivery in resource-scarce settings? Front. Psychiatry 11, 564452. 10.3389/fpsyt.2020.564452 33061919 PMC7518034

[B30] Tran B. X. Phan H. T. Nguyen T. P. T. Hoang M. T. Vu G. T. Thi Lei H.. (2020c). Reaching further by Village Health Collaborators: the informal health taskforce of Vietnam for COVID-19 responses. J. Glob. Health 10, 010354. 10.7189/jogh.10.010354 32509285 PMC7242882

[B31] Vietnam M. (2020). Against COVID-19 pandemic in Bach Mai Hospital. Available online at: https://moh.gov.vn/hoat-dong-cua-lanh-dao-bo/-/asset_publisher/k206Q9qkZOqn/content/don-luc-dap-dich-tai-benh-vien-bach-mai (accessed April 30, 2020).

[B32] Wenham C. Smith J. Morgan R. (2020). COVID-19: the gendered impacts of the outbreak. Lancet 395, 846–848. 10.1016/S0140-6736(20)30526-2 32151325 PMC7124625

[B33] WHO. (2020). Available online at: https://www.who.int/director-general/speeches/detail/director-general-s-opening-remarks-at-the-world-health-assembly (accessed May 24, 2021).

[B34] Worldometer (2020). COVID-19 coronavirus pandemic. Available online at: https://www.worldometers.info/coronavirus/ (accessed February 5, 2020).

[B35] Xue-Yan Zhang H.-J. H. Dong-Lin Z. Moussa Ide N. Ming-Hua Y. Ping Z. Ming-Yi Z. (2020). Biological, clinical and epidemiological features of COVID-19, SARS and MERS and AutoDock simulation of ACE2. Infect. Dis. Poverty 9, 99. 10.1186/s40249-020-00691-6 32690096 PMC7369569

[B36] Zhang Y. Ma Z. F. (2020). Impact of the COVID-19 Pandemic on mental health and quality of life among local residents in liaoning province, china: a cross-sectional study. Int. J. Environ. Res. Public Health 17:2381. 10.3390/ijerph17072381 32244498 PMC7177660

